# Risk factors for the occurrence of visual-threatening posterior capsule opacification

**DOI:** 10.1186/s12967-019-1956-6

**Published:** 2019-06-20

**Authors:** Hung-Chi Chen, Chia-Yi Lee, Chi-Chin Sun, Jing-Yang Huang, Hung-Yu Lin, Shun-Fa Yang

**Affiliations:** 1 Department of Ophthalmology, Chang Gung Memorial Hospital, Linkou, Taiwan; 2 Department of Medicine, Chang Gung University College of Medicine, Taoyuan, Taiwan; 3 Center for Tissue Engineering, Chang Gung Memorial Hospital, Linkou, Taiwan; 4 Department of Ophthalmology, Show Chwan Memorial Hospital, No. 2, Ln. 530, Sec. 1, Zhongshan Rd., Changhua City, 50093 Changhua County Taiwan; 5 Department of Optometry, College of Medicine and Life Science, Chung Hwa University of Medical Technology, Tainan, Taiwan; 6 Department of Ophthalmology, Chang Gung Memorial Hospital, Keelung, Taiwan; 7 Department of Chinese Medicine, Chang Gung University, Taoyuan City, Taiwan; 8 Department of Medical Research, Chung Shan Medical University Hospital, Taichung, Taiwan; 9 Institute of Medicine, Chung Shan Medical University, No. 110, Sec. 1, Chien-Kuo N. Rd., Taichung, 40201 Taiwan; 10 Department of Optometry, Chung Shan Medical University, Taichung, Taiwan; 11 Department of Exercise and Health Promotion, Chung Chou University of Science and Technology, Changhua, Taiwan

**Keywords:** Posterior capsule opacification, Cataract surgery, Nd:YAG capsulotomy, Intraocular lens, Population-based

## Abstract

**Background:**

To evaluate the potential risk factor of visual-threatening posterior capsule opacification (PCO) via the analysis of National Health Insurance Research Database in Taiwan.

**Patients and methods:**

A total of 8571 patients (3767 male and 4804 female) were recruited in the study group and 17,142 patients (7534 male and 9608 female) in the control group. Patients undergoing cataract surgery, acrysof single-piece intraocular lens implantation and Nd:YAG capsulotomy were selected as the study group. After exclusion which aimed to standardize the ocular condition and exclude the possibility that patients undergoing cataract surgery and Nd:YAG capsulotomy in different eyes, each patient in the study group was age-gender matched to two patients undergoing cataract surgery but without Nd:YAG capsulotomy. The demographic data, systemic disease, and ocular co-morbidities were obtained and analyzed. Adjusted odds ratio (OR) of each demographic data and co-morbidities to the development of visual-threatening PCO, and adjusted OR of co-morbidities to visual-threatening PCO develop within 1 year postoperatively.

**Results:**

The dry eye disease (DED), glaucoma, uveitis, age-related macular degeneration (AMD), hyperlipidemia, peptic ulcer disease and liver disease showed significant crude OR while the DED, glaucoma, AMD, hyperlipidemia and peptic ulcer disease revealed a significant adjusted OR. In the subgroup analysis, the DED, glaucoma, AMD, and hyperlipidemia still illustrated a higher adjusted OR to develop visual-threatening PCO within 1 year after the cataract surgery.

**Conclusion:**

The DED, glaucoma, AMD, hyperlipidemia and peptic ulcer disease may serve as the risk factor for the developing of visual-threatening PCO.

## Background

Cataract surgery has been well-developed and widely applied for decades with excellent visual outcomes [1]. However, several complications including posterior capsule tear, Descemet’s membrane detachment and vitreous loss may occur and lead to severe visual impairment [2]. Posterior capsule opacification (PCO) is one of the most common complications after cataract surgery which needs Nd:YAG capsulotomy to relieve the visual impairment for those visual-threatening cases [3, 4]. One known risk factor for developing PCO is the design and material of intraocular lens (IOL) for which the blunt edge would lead to a higher rate of PCO formation [3, 5, 6].

Concerning other potential risk factors for occurrence of PCO, uveitis of any subtype has been regarded as a prominent risk factor in which approximately 20 percent of patients with uveitis develop PCO within 1 year [7, 8]. On the other hand, diabetes mellitus (DM) was demonstrated as a protective factor for PCO with a significant lower rate [9]. However, there was rare research regarding the effect of other ocular and systemic diseases on the development of PCO. Since the possible pathophysiology of PCO included cell proliferation, transition and the activation of inflammatory mediators [10–12], it is possible that certain diseases that influence the intraocular condition may be the risk factors of PCO.

The purpose of the current study was to evaluate the potential risk factors of visual-threatening PCO via the analysis of National Health Insurance Research Database (NHIRD) in Taiwan. In addition, the potential risk factors leading to PCO development within 1 year after the cataract surgery were also investigated.

## Materials and methods

### Data source

This retrospective, population-based, nested case–control study was approved by the National Health Insurance Administration and the Institute Review Board of Chung Shan Medical University. Provided by the Taiwan National Health Research Institutes, the NHIRD contains insurance claims data of more than 99% of Taiwan’s population. The claims data used in this study were from the 2000 Longitudinal Health Insurance Database, which contains data on one million patients randomly sampled from the registry of the NHIRD for the year 2010. The 2000 Longitudinal Health Insurance Database data were linked from January 1, 1997, to December 31, 2013, and the International Classification of Diseases, ninth edition (ICD-9) codes as well as several procedure and surgery codes in NHIRD were used to identify the diseases. Medications prescribed, demographics, socioeconomic status and location of patients were also available in the NHIRD.

### Patient selection

Patients were regarded as having visual-threatening PCO if their medical records showed a diagnosis of cataract (ICD-9 codes: 366.10–366.19, 366.8, 366.9) and indicated an event of Nd:YAG capsulotomy (procedure code: 60013C) after cataract surgery (ICD-9 codes: V45.61, or surgery code: 86008C) from 1997 to 2013. Since the Nd:YAG capsulotomy was applied in patients whose visual acuity influence by PCO significantly in most cases, we regarded the performance of this procedure as the present of visual-threatening PCO. The index date was set as the date that patient received Nd:YAG capsulotomy and the date of enrollment was set as the date that patient received cataract surgery. To avoid possible confusion, only patients who visited ophthalmologists (department code: 10) and received acrysof single-piece IOL implantation (equipment code: SA60AT) were enrolled. In addition, the following exclusion criteria were applied to better standardize the ocular condition of each participants and exclude the possibility that patient received cataract surgery and Nd:YAG capsulotomy in different eyes: (1) having been diagnosed with legal blindness (ICD-9 code: 369.4); (2) having received any type of eyeball removal surgery (ICD-9 codes: 16.5x) before the index date; (3) having received an ocular tumor diagnosis (ICD-9 codes: 190.0–190.9) before the index date; (4) having received a diagnosis of major ocular trauma (ICD-9 codes: 870.x, 871.x, 921.x and 940.x) before the index date; (5) having received Nd:YAG capsulotomy before the enrollment date or before 2003; (6) having received cataract surgery (surgery code already mentioned above) or a diagnosis of post-cataract surgery status (ICD-9 codes: v43.1, v45.61 and v58.3) before the enrollment date; and (7) receive another cataract surgery (surgery code already mentioned above) between the enrollment date and the index date. In addition, each individual in the study group was age- and sex-matched to two individuals that received cataract surgery but no Nd:YAG capsulotomy was arranged which serve as the control group, and the index dates of those in the control group was corresponded with matched visual-threatening PCO patients. The index date was assigned dependent on the nested case–control design. Those visual-threatening PCO patients who could not be matched with two patients without visual-threatening PCO were excluded.

### Main outcome measurement

The following systemic comorbidities were retrospectively enrolled in the analysis model to survey the potential risk factor for visual-threatening PCO: hypertension (ICD-9 codes: 401.x-405.x), diabetes mellitus (DM) (ICD-9 codes: 250.x, 277.7), hyperlipidemia (ICD-9 codes 272.0, 272.1, 272.2, 272.4 and 272.9), congestive heart failure (ICD-9 codes: 398.91, 402.01, 402.11, 402.91, 404.01, 404.03, 404.11, 404.13, 404.91, 404.93, 425.4–425.9, 428.x), cerebrovascular disease (ICD-9 codes: 430.x-438.x), myocardial infarction (ICD-9 codes: 410.x-412.x), peripheral vascular disease (ICD-9 codes: 093.0, 437.3, 440.x, 441.x, 443.1–443.9, 47.1, 557.1, 557.9, V43.4), dementia (ICD-9 codes: 290.x, 294.1, 331.2), chronic pulmonary disease (ICD-9 codes: 416.8, 416.9, 490.x–505.x, 506.4, 508.1, 508.8), rheumatic disease (ICD-9 codes: 446.5, 710.0–710.4, 714.0–714.2, 714.8, 725.x), peptic ulcer disease (ICD-9 codes: 531.x–534.x), liver disease (ICD-9 codes: 070.22, 070.23, 070.32, 070.33, 070.44, 070.54, 070.6, 070.9, 456.0–456.2, 570.x, 571.x, 572.2–572.8, 573.3, 573.4, 573.8, 573.9, V42.7), hemiplegia or paraplegia (ICD-9 codes: 334.1, 342.x, 343.x, 344.0–344.6, 344.9), renal disease (ICD-9 codes: 403.01, 403.11, 403.91, 404.02, 404.03, 404.12, 404.13, 404.92, 404.93, 582.x, 583.0–583.7, 585.x, 586.x, 588.0, V42.0, V45.1, V56.x), malignancy including lymphoma (ICD-9 codes: 140.x–172.x, 174.x–195.8, 200.x–208.x, 238.6), and coagulation defects (ICD-9 codes: 286.x). For the ocular co-morbidities, keratopathy (ICD-9 codes: 370.0x, 370.2x, 370.3x, 370.4x, 370.5x, 370.6x, 370.8, 370.9, 371.0x, 371.21–371.23), dry eye disease (DED) (ICD-9 codes 370.33, 370.34, 372.53, 375.15), glaucoma (ICD-9 codes: 365.1x, 365.2x, 365.7x, 365.9), uveitis (ICD-9 codes: 360.12, 363.0x, 363.1x, 363.2x, 364.0x, 364.1x, 364.2x and 364.3) and age-related macular degeneration (AMD) (ICD-9 codes: 362.50, 362.51, 362.52) were enrolled in the analysis model. In addition, we also considered the effects of demographic conditions including urbanization and income level. To strengthen the time-chronicity between the potential risk factors and following visual-threatening PCO, only those pre-existing co-morbidities that emerged before the enrollment date were included in the analysis model.

### Statistical analysis

SAS version 9.4 (SAS Institute Inc, NC, USA) was employed for all the analyses. After age and sex matching, Chi square test was employed to test for differences in the demographic data (urbanization and income level) between the study and control groups. Then, the odds ratio (OR) with corresponding 95% confidence intervals (CI) and the crude OR were calculated by conditional logistic regression. In the next step, conditional logistic regression was adopted again but enrolled demographic data, prominent ocular diseases, and systemic comorbidities mentioned above in the multivariate model to compute adjusted OR of visual-threatening PCO, which were estimated to reduce the cofounding effects from demographic status, systemic comorbidities, and ocular diseases. In addition, the mean and median from enrollment date (cataract surgery) to index date (Nd:YAG capsulotomy) with each potential risk factors were yielded. For subgroup analysis, the patients in the study group were divided into those received Nd:YAG capsulotomy within 1 year after the cataract surgery and more than 1 year after the cataract surgery. Then, those significant risk factors that lead to the development of visual-threatening PCO were analyzed to yield the adjusted OR for the rapid-developing visual-threatening PCO that occurred within 1 year after cataract surgery, while the effects of all potential risk factors still enrolled in the multivariate model. Because most patients in the NHIRD are Han Taiwanese, race was not considered as a covariate. The results with *P *< 0.05 were regarded as statistically significant, and a *P* value of less than 0.0001 was depicted as *P *< 0.0001.

## Results

**Fig. 1:**
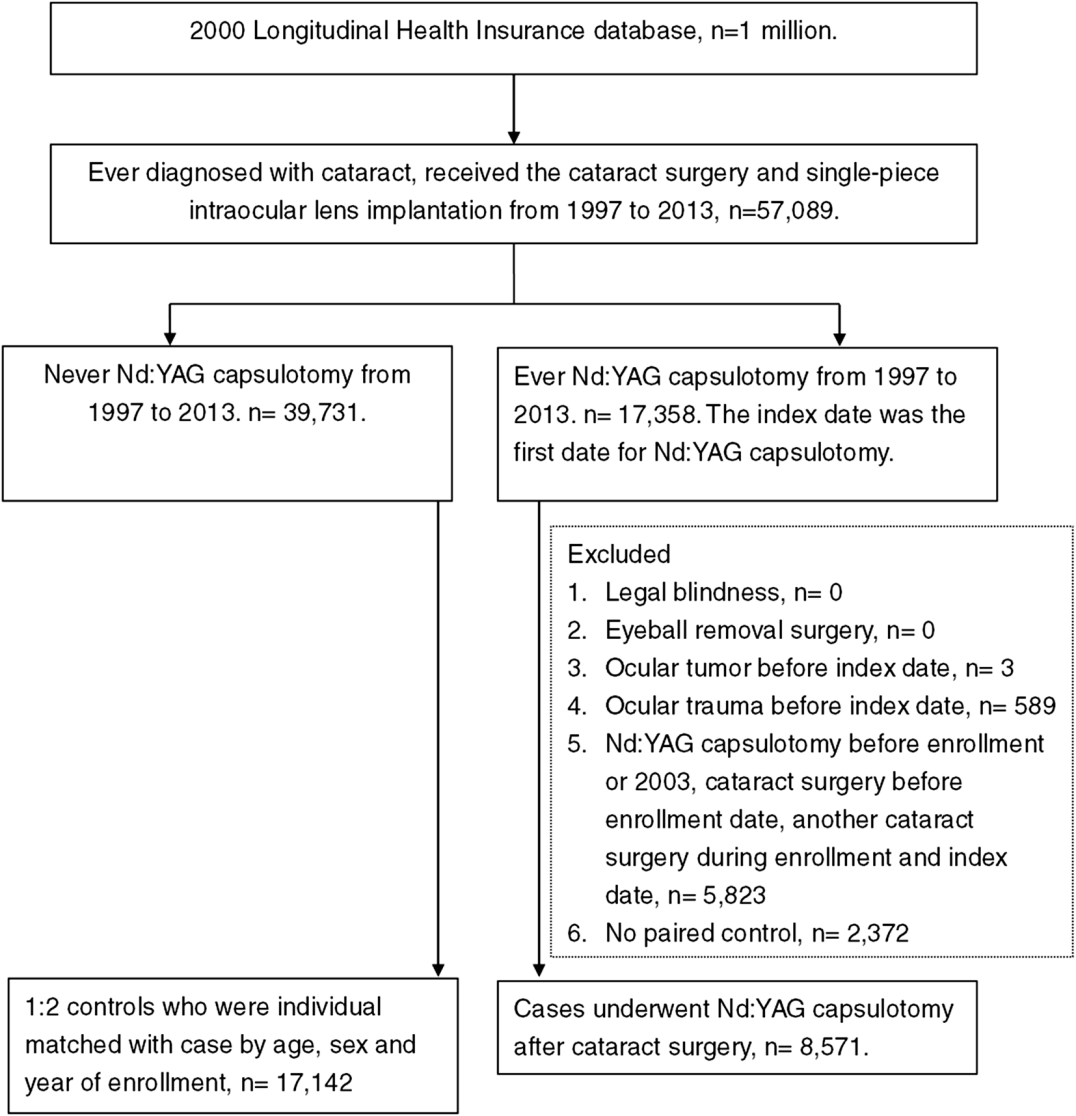
The flowchart of subject selection

The flowchart of subject selection was demonstrated in Fig. 1. After exclusion and matching, a total number of 8571 patients who received cataract surgery and Nd:YAG capsulotomy were enrolled in the study group while another 17,142 patients who received only cataract surgery without Nd:YAG capsulotomy were enrolled in the control group. The characteristics at baseline revealed an identical age and gender ratio due to matching, and a higher ratio of urbanization, DED, glaucoma, uveitis, AMD, hyperlipidemia, peptic ulcer disease and liver disease were found in the study group (Table 1).

**Table 1:** Characteristics in the study and control groups at baseline

	Control n = 17,142	Nd:YAG capsulotomy n = 8571	*P* value
Age at baseline, mean ± SD	72.17 ± 8.74	72.17 ± 8.74	1.0000
< 40	6 (0.04%)	3 (0.04%)	
40–59	1552 (9.05%)	776 (9.05%)	
60–79	12,030 (70.18%)	6015 (70.18%)	
≥ 80	3554 (20.73%)	1777 (20.73%)	
Gender			1.0000
Female	9608 (56.05%)	4804 (56.05%)	
Male	7534 (43.95%)	3767 (43.95%)	
Urbanization			< 0.0001
Urban	8921 (52.04%)	4702 (54.86%)	
Sub-urban	5331 (31.1%)	2620 (30.57%)	
Rural	2890 (16.86%)	1249 (14.57%)	
Low income	84 (0.49%)	34 (0.40%)	0.2965
Co-morbidities			
Corneal disease	1674 (9.77%)	868 (10.13%)	0.3597
DED	1783 (10.4%)	1010 (11.78%)	0.0008
Glaucoma	1768 (10.31%)	1078 (12.58%)	< 0.0001
Uveitis	180 (1.05%)	115 (1.34%)	0.0384
AMD	876 (5.11%)	510 (5.95%)	0.0049
Hypertension	9585 (55.92%)	4769 (55.64%)	0.6764
DM	5138 (29.97%)	2449 (28.57%)	0.0203
Hyperlipidemia	4739 (27.65%)	2516 (29.35%)	0.0041
Congestive heart failure	1462 (8.53%)	738 (8.61%)	0.8253
Myocardial infarction	308 (1.8%)	145 (1.69%)	0.5463
Cerebrovascular disease	2414 (14.08%)	1215 (14.18%)	0.8394
Peripheral vascular disease	833 (4.86%)	435 (5.08%)	0.4511
Dementia	408 (2.38%)	161 (1.88%)	0.0099
Chronic pulmonary disease	4363 (25.45%)	2231 (26.03%)	0.3174
Rheumatic disease	470 (2.74%)	241 (2.81%)	0.7469
Peptic ulcer disease	4274 (24.93%)	2375 (27.71%)	< 0.0001
Liver disease	2830 (16.51%)	1517 (17.7%)	0.0164
Hemiplegia or paraplegia	347 (2.02%)	146 (1.7%)	0.0770
Renal disease	1470 (8.58%)	665 (7.76%)	0.0253
Malignancy including lymphoma	1084 (6.32%)	540 (6.3%)	0.9422
Coagulation defects	28 (0.16%)	12 (0.14%)	0.6545

*SD* standard deviation, *DED* dry eye disease, *AMD* age-related macular degeneration, *DM* diabetes mellitus

After the conduction of conditional logistic regression, the DED, glaucoma, uveitis, AMD, hyperlipidemia, peptic ulcer disease and liver disease showed significant crude OR in the study group compared to the control group. And after adjustment for all potential risk factors, the DED, glaucoma, AMD, hyperlipidemia and peptic ulcer disease revealed a significant adjusted OR in the study group (Table 2). In addition, the mean and median time interval to receive Nd:YAG capsulotomy after cataract surgery of the above risk factors were lower compared to the general interval in the study group (Table 3).

**Table 2:** Odds ratio of Nd:YAG capsulotomy in patients with Nd:YAG capsulotomy after cataract surgery

Variables	Crude OR	Adjusted OR
Urbanization		
Urban	Reference	Reference
Sub-urban	0.93 (0.877–0.987)	0.935 (0.881–0.992)
Rural	0.817 (0.757–0.881)	0.821 (0.760–0.886)
Low income	0.807 (0.54–1.205)	0.803 (0.536–1.202)
Co-morbidities		
Keratopathy	1.042 (0.955–1.137)	0.988 (0.904–1.081)
DED	1.16 (1.066–1.262)	1.114 (1.022–1.215)
Glaucoma	1.251 (1.154–1.357)	1.222 (1.125–1.326)
Uveitis	1.281 (1.013–1.621)	1.185 (0.933–1.505)
AMD	1.177 (1.051–1.319)	1.147 (1.024–1.286)
Hypertension	0.988 (0.936–1.043)	0.976 (0.92–1.034)
DM	0.933 (0.881–0.989)	0.905 (0.85–0.963)
Hyperlipidemia	1.092 (1.03–1.158)	1.107 (1.039–1.179)
Congestive heart failure	1.011 (0.921–1.109)	1.024 (0.929–1.128)
Myocardial infarction	0.94 (0.77–1.148)	0.927 (0.756–1.135)
Cerebrovascular disease	1.008 (0.935–1.086)	1.02 (0.943–1.103)
Peripheral vascular disease	1.047 (0.929–1.179)	1.035 (0.917–1.168)
Dementia	0.786 (0.654–0.944)	0.762 (0.632–0.918)
Chronic pulmonary disease	1.032 (0.971–1.096)	1.014 (0.953–1.079)
Rheumatic disease	1.027 (0.876–1.203)	0.996 (0.849–1.168)
Peptic ulcer disease	1.158 (1.091–1.229)	1.156 (1.087–1.229)
Liver disease	1.087 (1.015–1.164)	1.067 (0.993–1.145)
Hemiplegia or paraplegia	0.839 (0.690–1.020)	0.828 (0.679–1.009)
Renal disease	0.894 (0.812–0.985)	0.876 (0.793–0.968)
Malignancy	0.996 (0.895–1.109)	0.974 (0.874–1.085)
Coagulation defects	0.857 (0.436–1.686)	0.883 (0.447–1.744)

*OR* odds ratio, *DED* dry eye disease, *AMD* age-related macular degeneration, *DM* diabetes mellitus

**Table 3:** The time interval from enrollment date to index date of each co-morbidity in the study group

	Time interval (months)	
	Mean (SD)	Median (Q1, Q3)
All Nd:YAG capsulotomy	32.12 (31.19)	25 (7, 45)
Co-morbidities		
Corneal disease	26.32 (27.18)	20 (5, 37)
DED	22.77 (22.65)	17 (5, 34)
Glaucoma	26.71 (27.58)	19 (5, 38)
Uveitis	21.03 (24.87)	13 (3, 30)
AMD	23.31 (22.96)	16 (4, 36)
Hypertension	28.02 (26.24)	22 (6, 41)
DM	27.73 (27.17)	20 (6, 41)
Hyperlipidemia	25.8 (24.71)	19 (5, 38)
Congestive heart failure	26.55 (24.69)	20 (6, 41)
Myocardial infarction	31.62 (30.11)	21 (6, 44)
Cerebrovascular disease	27.47 (26.5)	20 (6, 39)
Peripheral vascular disease	27.97 (27.38)	20 (6, 41)
Dementia	24.3 (27.37)	14 (3, 35)
Chronic pulmonary disease	29.14 (26.74)	23 (7, 43)
Rheumatic disease	28.49 (24.5)	25 (8, 43)
Peptic ulcer disease	28.28 (26.66)	22 (7, 41)
Liver disease	28.71 (26.51)	23 (7, 42)
Hemiplegia or paraplegia	24.53 (25.98)	16 (5, 37)
Renal disease	26.22 (25.26)	20 (6, 39)
Malignancy	25.7 (23.84)	20 (6, 39)
Coagulation defects	34.92 (38.05)	22 (7, 51.5)

*SD* standard deviation, *DED* dry eye disease, *AMD* age-related macular degeneration, *DM* diabetes mellitus

In the subgroup analysis to evaluate the risk factors that associated with a visual-threatening PCO within 1 year after the cataract surgery, the DED, glaucoma, AMD, and hyperlipidemia still illustrated a higher adjusted OR while the peptic ulcer disease showed non-significant result. There was also no influence of urbanization and income level, and the details were showed in Table 4.

**Table 4:** Odds ratio of receiving Nd:YAG capsulotomy within 1 year after the cataract surgery in the study group

Variables	Adjusted OR
Urbanization	
Urban	Reference
Sub-urban	0.960 (0.867–1.065)
Rural	0.799 (0.696–0.918)
Low income	0.9 (0.432–1.876)
Co-morbidities	
DED	1.504 (1.311–1.727)
Glaucoma	1.239 (1.083–1.418)
AMD	1.432 (1.191–1.723)
Hyperlipidemia	1.212 (1.09–1.347)
Peptic ulcer disease	1.086 (0.978–1.206)

*OR* odds ratio, *DED* dry eye disease, *AMD* age-related macular degeneration

## Discussion

Briefly, the current study showed an increased risk for the visual-threatening PCO in patients with preceding DED, glaucoma, AMD, hyperlipidemia and peptic ulcer disease. On the other hand, the DED, glaucoma, AMD and hyperlipidemia would elevate the possibility to develop such type of PCO within 1 year postoperatively. The results were correlated to the shorter time interval from cataract surgery to the development of visual-threatening PCO in patients with those risk factors.

Several mechanisms have been proposed for the development of PCO. One of the important pathophysiology is the migration and epithelial-mesenchymal transition of lens epithelial cells (LECs) [10]. The LECs may spread into the anterior chamber and the capsule bag during cataract surgery, proliferating and transdifferentiating into myofibroblastic cells, and finally form fibrotic plaques on implanted IOL and end up with PCO [10]. Another pathway for PCO is the activation of intraocular macrophages after surgery, in which macrophages aggregate at the posterior capsule and IOL in patients with PCO [12, 13]. In addition, certain cytokines like epidermal growth factor, matrix metalloproteinases and interleukins are related to such process [10, 11, 14, 15]. In previous experimental studies, interleukin-6 which can be produced by LECs was found in other fibrotic ocular diseases and fibrous tissue of PCO [14]. On the other hand, the lipid component may also be linked to PCO since lipid peroxidation may lead to the dysfunction of LECs and the formation of cataract [16]. Accordingly, PCO may be correlated to diseases involving aforementioned pathways and several co-morbidities were found to be related to the visual-threatening PCO in the current study.

In the current study, the ocular diseases correlated to the development of visual-threatening PCO including DED, glaucoma and AMD. To our knowledge, this is a preliminary experience to demonstrate these pre-existing ocular diseases as a risk factor for the developing of visual-threatening PCO. Moreover, these three ocular diseases also associated with the rapid-onset visual-threatening PCO which occurred only 1 year after the cataract surgery which further strengthened the correlation. Although the definitive pathophysiology of DED is still in investigation, evidence has shown that the inflammatory process plays a major role in the course of DED [17], probably leading to the development of PCO [11]. Although both open angle and close angle subtypes of glaucoma were included in the statistical analysis, It is possible that elevated intraocular pressure not only damage the retinal nerve fiber layer but also result in the impairment of capsule structure and cell biology of LECs. For the retinal disorders, the immune-inflammatory reactions would contribute to the dry and wet type AMD according to previous studies [18, 19]. Similarly, the intraocular inflammatory activation are related to the LECs migration and the formation of PCO [20]. Still, whether treatments targeting on DED, glaucoma and AMD influence the formation of visual-threatening PCO merits further investigation.

Interestingly, the pre-existing uveitis did not correlated to the development of visual-threatening PCO after adjusting other demographic data and co-morbidities in the multivariate model. The current finding is opposite to the established concept that PCO is a common complication in patient diagnosed with uveitis in which the occurrence rate could up to 58 percent [7, 8, 21]. There are some possible explanations for the conflicting results. First, the mean adjusted OR of uveitis is 1.185, similar to the other three ocular diseases (i.e. DED, glaucoma and AMD). On the other hand, both the mean and median time intervals from cataract surgery to Nd:YAG capsulotomy in patients with uveitis were the shortest among all the ocular as well as systemic co-morbidities, implying a rapid-developing visual threatening PCO is common with pre-existing uveitis. Moreover, patients with uveitis usually develop cataract earlier than others due to the application of topical steroid, causing exclusion of such patients from the current study because of the failure of age-gender match to patients without visual-threatening PCO. We speculate that the pre-existing uveitis is still a significant risk factor for the visual threatening PCO which already showed marginal significance in the current study.

Concerning the systemic diseases that illustrated as a risk factor of visual-threatening PCO, both the hyperlipidemia and peptic ulcer disease showed significant OR after adjustment in the multivariate model. The hyperlipidemia is a chronic status of lipid component dysregulation and has been proven to be associated with ocular diseases like meibomian gland dysfunction and blepharitis [22, 23]. The persistent dyslipidemia may also affect the intraocular lipid precipitation and lipid perioxidation [16], thus accelerate the formation of PCO. For the peptic ulcer disease, the cytokine simulated in the peptic ulcer disease including interleukins identical to those immune mediators observed in the PCO [14, 24]. In the subgroup analysis, the preceding hyperlipidemia was a risk factor for the visual-threatening PCO which occurred 1 year postoperatively while such relationship did not found from the patient with peptic ulcer. Since hyperlipidemia is an asymptomatic disease [25], a longer disease interval and effect may appear in patient with hyperlipidemia than peptic ulcer disease.

Up to now, the confirmed risk factors for the development of PCO are the design and material of IOL [26]. A sharper edge prevents cell migration onto the posterior surface of IOL, while hydrophilic materials lead to a higher rate of PCO occurrence than hydrophobic ones [26, 27]. And in a previous study, the multifocal design of IOL resulted in higher PCO rate compared to the monofocal type [28]. In the current study, only patients implanted with acrysof single-piece IOL were included so the effect of different types of IOL can be neglected. There were 8571 patients receiving Nd:YAG capsulotomy (after exclusion of inappropriate cases) from 57,089 patients receiving cataract surgery and the mean time interval from cataract surgery to Nd:YAG capsulotomy was 32.12 ± 31.19 months, similar to a previous study using similar IOL in which 21.7% patients needed Nd:YAG capsulotomy for PCO 3 year postoperatively [29].

There are still some limitations in the current study. First, the retrospective nature and case–control design may diminish the power of the current study. Second, the different subtypes of co-morbidity (i.e. dry and wet type AMD) have not been evaluated in the current study due to the difficulty on analysis which may lead to bias. Third, since various premium IOL in Taiwan including aspheric, toric, multifocal and segmental designs are not reimbursed by the National Health Insurance Administration, the effect of IOL design could not be evaluated. Furthermore, it is possible that some patients receiving cataract surgery are not covered by the National Health Insurance Administration, which may result in bias. However, the chance for this condition is rare according to the clinical experience.

## Conclusions

In conclusion, the DED, glaucoma, AMD, hyperlipidemia and peptic ulcer disease may be considered as risk factor for the developing of visual-threatening PCO after adjusting for available confounders. Moreover, the DED, glaucoma, AMD and hyperlipidemia are likely to correlate to a rapid-onset visual-threatening PCO that occur within 1 year after the cataract surgery. Further large-scale prospective study to investigate the effectiveness of different disease interval, pathophysiology and IOL design on the developing of visual-threatening PCO is mandatory.

## Data Availability

All data generated or analysed during this study are included in this published article and its additional files.

## Abbreviations

AMD: age-related macular degeneration
DED: dry eye disease
IOL: intraocular lens
NHIRD: National Health Insurance Research Database
PCO: posterior capsule opacification
OR: odds ratio
